# Rational Integration of ZIF-8 and BiPO_4_ for Energy Storage and Environmental Applications

**DOI:** 10.1021/acsomega.2c04835

**Published:** 2022-11-28

**Authors:** Sevda Yetiman, Sultan Karagoz, Fatma Kilic Dokan, M. Serdar Onses, Erkan Yilmaz, Ertugrul Sahmetlioglu

**Affiliations:** † ERNAM-Erciyes University Nanotechnology Application and Research Center, Kayseri38039, Turkey; ‡ Department of Textile Engineering, Faculty of Engineering, Erciyes University, Kayseri38039, Turkey; § Department of Chemistry and Chemical Processing Technologies, Mustafa Çıkrıkcıoglu Vocational School, Kayseri University, Kayseri38280, Turkey; ∥ Department of Materials Science and Engineering, Faculty of Engineering, Erciyes University, Kayseri38039, Turkey; ⊥ Technology Research & Application Center (TAUM), Erciyes University, Kayseri38039, Turkey; # Department of Basic Sciences of Engineering, Kayseri University, Kayseri38039, Turkey; ∇ Department of Analytical Chemistry, Faculty of Pharmacy, Erciyes University, Kayseri38280, Turkey

## Abstract

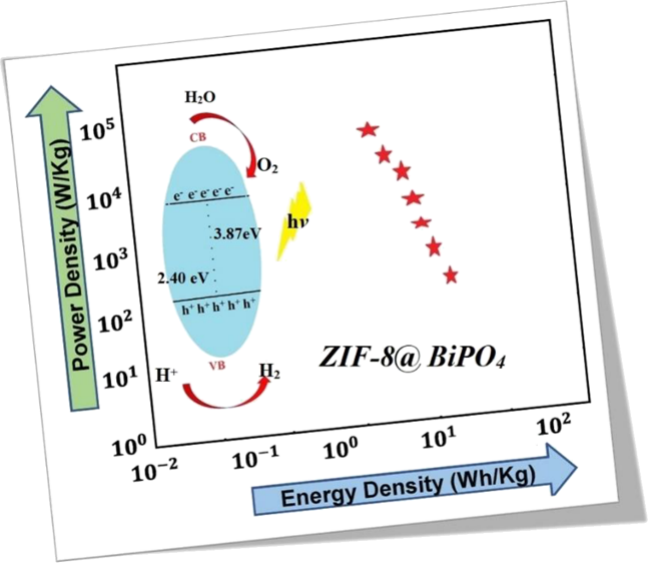

Environmental pollution and energy storage are among the most pivotal challenges of today’s world. The development of multifunctional materials is required to address these challenges. Our study presents the rational design and synthesis of a hybrid material (ZIF-8@BiPO_4_) with dual functionality: an outstanding supercapacitor electrode and an excellent photocatalyst. The ZIF-8@BiPO_4_ hybrid structure was obtained by conjoining zinc ions and 2-methylimidazole ligands toward BiPO_4_ by a one-pot stirring route at room temperature. The ZIF-8@BiPO_4_ resulted in considerably higher specific capacitance (Cs) (489 F g^–1^ at a scan rate of 5 mV s^–1^; 497 F g^–1^ at a current density of 1 A g^–1^) than that of pure BiPO_4_ (358; 443 F g^–1^) and ZIF-8 (185; 178 F g^–1^) under the same conditions in a three-electrode cell using the 2 M KOH aqueous electrolyte. Afterward, an asymmetric supercapacitor (ASC) device was fabricated with BiPO_4_ as the anode and ZIF-8@BiPO_4_ as the cathodes, acquiring an outstanding Cs of 255 F g^–1^ at a current density of 0.5 A g^–1^ with significant cycling stability (81% over 10,000 cycles). Moreover, the ASC has an energy density of 17.5 Wh kg^–1^and a power density of 13,695 W kg^–1^, which can be considered to be at the borderline between batteries and supercapacitors. The photocatalytic activity of ZIF-8@BiPO_4_ was further studied using a methylene blue (MB) dye and sildenafil citrate (SC) drug-active molecules. The degradation of MB was approximately 78% through the photocatalytic reduction after 180 min of UV irradiation. The outstanding characteristics together with the ecofriendly and low-cost preparation make ZIF-8@BiPO_4_ appealing for a broad range of applications.

## 1 Introduction

Clean energy and clean water are among the 17 sustainable development goals set up by the United Nations General Assembly.^1^ The rapid industrialization and information age particularly have detrimental effects on both of these challenges. Technology-oriented life accompanied many conveniences besides some formidable environmental problems. Within that period, innovatory yet low-impact approaches to energy storage accentuate the importance of energy conservation with contamination-free materials. Among the energy storage devices, supercapacitors (SCs) have distinctive features as they have almost unlimited life cycle, economical construction, high power density, and swift charge–discharge process, besides boosted safety compared with batteries and fuel cells.^2−8^

A typical SC transpires three sorts of materials, which are an electrode, an electrolyte, and a current collector. While all chemical reactions and physical phenomena at the electrode/electrolyte surface are taken into consideration, electrode materials play a pivotal role in forming high-quality SCs. Novel hybrid materials with high energy and power densities are required for the practical utilization of SCs. In that case, asymmetric supercapacitors (ASCs) or hybrid SCs arouse interest with a boosted potential window (0–2 V) when compared with symmetric supercapacitors (0–1 V). This brings forth the need for a sweeping type of material.^9−15^

In particular, graphene discovery highlighted the nanocarbon-based material’s irreplaceable impact on energy storage.^10,16−18^ By this time, there have been many studies on the singly or doped effect of activated carbon (AC), graphene, graphene oxide, carbon nanotubes (CNTs), graphical carbon nitride (g-C_3_N_4_), carbon dots (CDs), and carbon quantum dots.^19−35^ Transition metal oxides, metal sulfides, polyoxometalates, phosphates, carbonates, chalcogenides, metal–organic frameworks (MOFs) conductive polymers, and their compounds have been investigated as adequate materials for energy storage applications, and they have a domain in other areas such as catalysts, sensors, drug delivery, gas storage/separation, and other types of energy storage/conversion systems such as batteries and fuel cells.^36−47^ Chiefly, metal oxides including Co_3_O_4_, Fe_2_O_3_, V_2_O_5_, RuO_2_, ZnO, MnO_2_, NiO, SnO_2_, TiO_2_, CuO, Bi_2_O_3_, etc. with high specific capacitance values and high pseudocapacitive behaviors are commonly used.^35,48−63^ Among the available metal oxides, Co_3_O_4_ and RuO_2_ come to the forefront with magnificent electrochemical reversibility and high theoretical specific capacitance, as well as remarkable cycling stability.^60^ However, the steep price and toxic nature of these materials limit their utilizability.^64,65^ To sum up, the formation of ideal SCs requires reasonably priced and environmentally friendly materials with high capacitance, besides high energy and power densities.

Recently, BiPO_4_ materials have been widely utilized and have attracted substantial attention thanks to their ecofriendly, atoxic essence, and dielectric behavior caused by high oxygen conductivity.^66−68^ The ability to be synthesized in different sizes with various morphologies such as spherical, octahedral, or rod is also a vantage of BiPO_4_, which enhanced its utilization in SCs and photocatalytic applications. For photocatalytic applications, the use of phosphate-containing photocatalysts, which have tremendous properties such as high electronegativity of anions that promote electron and hole separation, high crystallinity, high photocatalytic oxidation performance, and good stability, has gained an important place in the literature. One of the parameters affecting the photocatalytic performance of BiPO_4_ photocatalysts is the crystal phase of the photocatalysts. For example, it has been discovered that the hexagonal phase of BiPO_4_ showed a lower photocatalytic performance than the monoclinic phase of BiPO_4_.^68,69^

The competent and reversible redox reactions of Bi^3+^ ions and the characteristic idiosyncrasy stability of the phosphate groups engender BiPO_4_ to demonstrate exquisite electrochemical performance. Particularly, its redox peak position in the voltage range of carbon materials (−1 to 0 V in aqueous electrolytes) utterly pointed out that it would be more feasible to be an alternative for carbon-based materials in ASCs.^70,71^

Yet, other cutting-edge materials are MOFs due to their unique structural features such as open metal sites, high surface areas, tailored pore sizes, ultralow density, and postsynthetic modifications.^72−74^ Zeolitic imidazolate frameworks (ZIFs) are a subdivision of MOFs designed via the correlation of metal ion notes (like zinc or cobalt) with an organic binder in a convenient solvent. As a principle, an electronic channel is supplied by the oxidation–reduction of metal ions while the charge transfer in the framework is assisted via the linker.^75^ Especially, the ZIF-8 (formed by zinc ions with the linkage of 2-methylimidazole) has excellent properties such as a large surface area (BET, 1413 m^2^ g^–1^), with a large pore size (diameter of 11.6 Å), good thermal stability (up to 550 °C), and significant chemical resistance to boiling alkaline water and organic solvents.^76,77^ With these multifaceted properties, it can be considered an alternative material to TiO_2_ and ZnO for photocatalytic applications.^76^

Studies in the literature not only show that porous ZIF materials are a potential new photocatalyst class, but also trigger extensive research on ZIF photocatalysts. Compared with traditional semiconductor photocatalysts such as ZnO, TiO_2_, and BiWO_4_, photoactive ZIFs have significant utility advantages in photocatalytic applications: (i) the well-defined crystal structures of ZIFs are useful in the study and characterization of the structure–property relationship of these solid photocatalysts; (ii) the modular nature of the ZIF synthesis allows for the rational design and fine-tuning of these catalysts at the molecular level, allowing for easy tuning of the electronic structure of ZIF catalysts; (iii) structural features of tunable active sites (i.e., metal-oxoclusters and organic binders) in ZIFs pave the way for more efficient use of photon energy; (iv) unlike typical traditional metal oxide photocatalysts, visible light photocatalytic activity can be readily introduced via linker substitutions of organic chromophores in ZIF structures like amino groups.^76,77^ However, poor conductivity and less cycling stability of ZIF-8 confine its usage in SCs. Thereafter, ZIFs are mainly utilized to obtain nitrogen (N)-doped porous carbon electrode materials.

To date, increasing interest has been centered on BiPO_4_ and ZIF-8 electrode materials, and some crucial advancement has been performed. For instance, a comparison between AC and BiPO_4_ was made by Wang et al.^78^ They reported a maximum specific capacitance of AC 117.3 F g^–1^ at a current density of 1 A g^–1^, while the capacity of BiPO_4_ was 360.56 F g^–1^ at the same current density and under the same potential window. Vadivel and co-workers examined a BiPO_4_/MWCNT (1D-1D) composite structure synthesized by a solvothermal route. The recorded maximum specific capacitance was 504 F g^–1^ at a scan rate of 5 mV s^–1^. Moreover, they investigated the photocatalytic activity of the materials by using methyl orange (MO) as a target pollutant and notified that the BiPO_4_/MWCNT composite presented improved photocatalytic activity compared to pure BiPO_4_ under UV light irradiation.^66^ Nithya et al. examined various pH effects on the BiPO_4_ syntheses under various irradiation times and ultrasonication powers. A maximum specific capacitance of 1052 F g^–1^ (pH = 7 at 2 mV s^–1^) was obtained for the BiPO_4_ formed in the 2 h irradiation time with 60% power.^79^ Gao et al. scrutinized SnO_2_ quantum dots@ZIF-8 electrochemical features as a pseudocapacitor material. The recorded maximum specific capacitance of the composite was 931 F g^–1^ at a sweep rate of 5 mV s^–1^, while pure materials SnO_2_ quantum dots, and ZIF-8 just had a specific capacitance of 241 and 99 F g^–1^, respectively.^80^

Herein, we developed a new approach to enhance the electrochemical properties of ZIF-8 addedly environmental consciousness of BiPO_4_. The ZIF-8@BiPO_4_ hybrid structure was formed by compiling zinc ions and 2-methylimidazole ligands toward BiPO_4_ by the one-pot stirring method at room temperature. Thuswise, the electrical conductivity of the composite was improved by dispersing BiPO_4_ homogeneously within the ZIF-8 matrix. The ZIF-8@BiPO_4_ hybrid structure with exquisite features not only served as an excellent supercapacitor cathode, resulting in high energy and power densities but also was an outstanding photocatalysis with high methylene blue (MB) dye degradation. The results show that the ZIF-8@BiPO_4_ hybrid has a higher specific capacitance of 497 F g^–1^ than pure ZIF-8 (178 F g^–1^) and BiPO_4_ (443 F g^–1^) at a current density of 1 A g^–1^ in the three-electrode system. Furthermore, the modeled ASC device implementing a ZIF-8@BiPO_4_ cathode electrode and a BiPO_4_ anode electrode could reach a working voltage of 1.4 V, exhibiting a maximum power density of 7067 W kg^–1^ with a maximum energy density of 17.5 Wh kg^–1^. Meanwhile, the photocatalytic activity of the ZIF-8@BiPO_4_ (78%) hybrid was reported to be higher than that of pure BiPO_4_ (15%) thanks to the ZIF-8 (81%) excellent photocatalytic feature. Consequently, this multifunctional hybrid material with high photocatalytic activity and excellent electrochemical behaviors can be considered an example of a promising material for energy storage and water remediation applications.

## 2 Experimental Section

### 2.1 Materials

Bismuth nitrate pentahydrate (Bi(NO_3_)_3_·5H_2_O), potassium hydroxide (KOH), nitric acid (HNO_3_), sodium phosphate monobasic dihydrate (NaH_2_PO_4_·2H_2_O), zinc nitrate hexahydrate (Zn(NO_3_)_2_)·6H_2_O), sodium dodecyl sulfate (NaC_12_H_25_SO_4_)(SDS), and methanol (CH_3_OH) were merchandized from Merck (Germany). 2-Methylimidazole (C_4_H_6_N_2_), polyvinylidene fluoride, and *n*-methyl-2-pyrrolidone (NMP) were purchased from Sigma-Aldrich (USA). All chemical reagents and solvents for synthesis and analysis were utilized without further purification, and the used deionized water (DI) had 18.2 MΩ cm resistivity.

### 2.2 Synthesis of ZIF-8

The synthesis procedure of ZIF-8 was similar, with some small modifications reported by Bustamante et al.^81^ In detail, 4 mmol Zn(NO_3_)_2_)·6H_2_O was dissolved in 20 mL of CH_3_OH solution homogeneously. In another beaker, 32.3 mmol C_4_H_6_N_2_ was also dissolved in 20 mL of CH_3_OH with ultrasonication for 10 min. Thereafter, these two different solutions were mixed and magnetically stirred for 8 h. White ZIF-8 powders were obtained by washing three times with methanol and DI and vacuum-dried at 85 °C for 15 h.

### 2.3 Synthesis of BiPO_4_

The approach of modification of BiPO_4_ was carried out as previously reported. First, 0.5 g of NaH_2_PO_4_·2H_2_O was dissolved in 50 mL of DI until a homogeneous solution was observed. Then, 5 mmol SDS was added to the solution, and the compound was ultrasonically irradiated for 15 min. In another beaker, 5 mmol Bi(NO_3_)_3_·5H_2_O was in DI and 1 M HNO_3_ added to the solution until pH = 10. Later, the obtained white homogeneous solution was hydrothermally treated for 24 h at 180 °C. Then, the hydrothermally treated solution was cooled down to room temperature and centrifuged after washing DI and ethanol to remove impurities. As-obtained white particles were vacuum-dried at 85 °C for 12 h and saved in a desiccator for further usage.^79^

### 2.4 Synthesis of ZIF-8@BiPO_4_

**Figure 1:**
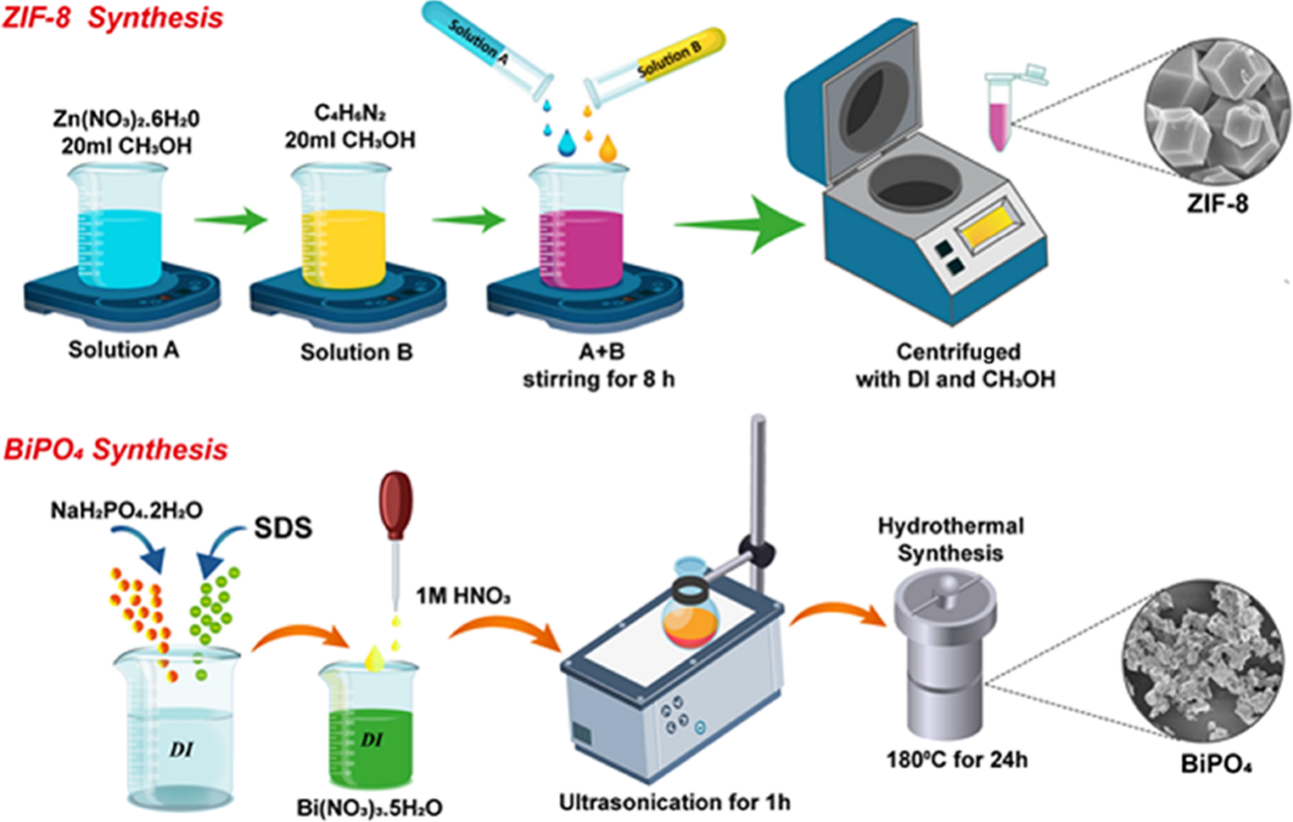
Synthesis steps of ZIF-8 and BiPO_4_.

The composite ZIF-8@BiPO_4_ was implemented by adding 200 mg of as-prepared BiPO_4_ to the mixture of 20 mL of C_4_H_6_N_2_/CH_3_OH solution. The rest of the synthesis process was similar to that for pure ZIF-8. Figure 1 shows the schematic illustration of the synthesis process.

### 2.5 Structural Characterization

To inspect the crystallographic structures of the patterns, a Bruker AXS D8 X-ray powder diffractometer with a simple cubic lattice and CU Kα radiation (λ = 0.15406 nm) was used, and the scan range (2θ) changing 5–90° was implemented. Fourier transform infrared spectroscopy (FTIR, Spectrum Two, PerkinElmer, USA) was applied to probe the structure and chemical bonds of the molecule. The Raman spectrum was investigated using a Raman microscope (Alpha 300 M+, WITec, Germany). The morphology of the patterns was screened using a field-emission scanning electron microscope (FESEM, Gemini 550) and a scanning transmission electron microscope (STEM, Gemini 550). Brunauer–Emmett–Teller (BET) and Barrett–Joyner–Halenda methods were used to analyze the pore size distribution, total pore volume (TPV), and the specific surface area (SSA) of the patterns.

### 2.6 Electrochemical Characterization

To obtain the working electrodes the electroactive materials (BiPO_4_, ZIF-8, and BiPO_4_@ZIF-8): 85 wt %, acetylene black: 10 wt % and PDVF: 5 wt % were intermingled with the inclusion of a few drops of the NMP solvent. The chosen current collector was a nickel foam in the dimensions of 1 cm × 1 cm. The oxide layer on the nickel foam was removed by cleaning 1 M HCl. The electrode was obtained by dropping the slurry on the nickel foam and drying at 85 °C for 12 h under vacuum. Roughly 1.1 mg mass of the active material was acquired.

A Gamry Reference 3000 electrochemical workstation was used to test the electrochemical measurements of the patterns in a typical three-electrode configuration by cyclic voltammetry (CV), galvanostatic charge–discharge (GCD), and electrochemical impedance spectroscopy (EIS). The electroactive materials were operated as working electrodes, Pt foil (1 × 1 cm^2^) as the counter electrode in which the reference electrode was silver/silver chloride (Ag/AgCl). All electrochemical analysis and measurements were conducted in 2 M KOH aqueous electrolyte.

The ASC device was formed by using BiPO_4_ as the anode and ZIF-8@BiPO_4_ as the cathode with a glass microfiber (Whatman) fiber as a separator. The CV, GCD, EIS, and the long-term test measurements of the asymmetric device were performed in the voltage range of 0–1.4 V.

### 2.7 Photocatalytic Degradation Studies on ZIF-8@BiPO_4_

Photocatalytic degradation experiments were carried out by using MB dye and sildenafil citrate (SC) as model pollutants under ultraviolet (UV) irradiation. The degradation of MB or SC was performed by exposure to a UV irradiation source (400 W UV lamp at λ = 380 nm) from the top of the reactor. For each experiment, 100 mg of the catalyst was added into a glass beaker containing 100 mL of prepared MB or CS aqueous solution at a concentration of 10 mg L^–1^. To establish an adsorption–desorption equilibrium between the pollutant and the photocatalyst, the prepared solution was magnetically stirred in the dark for 60 min and then subjected to UV light for 180 min. During UV exposure, MB or CS solution (1 mL) with a catalyst at certain time intervals was withdrawn and then centrifuged to remove the solid catalyst for analysis. Thereafter, the concentration of MB in the solution was measured by UV–vis absorption spectroscopy at 664 nm and CS concentration in the solution was measured by the *ultraperformance liquid chromatography method* with diode array detection (UPLC-DAD).

Finally, the percentage degradation of MB was calculated by eq 1:
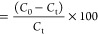
1where *C*_0_ (mg L^–1^) is the initial equilibrium concentration of MB at 664 nm and *C*_t_ (mg L^–1^) is the concentration of MB at 664 nm at the given time “*t*” min.^82^

## 3 Results and Discussion

### 3.1 Structural and Morphological Studies

**Figure 2:**
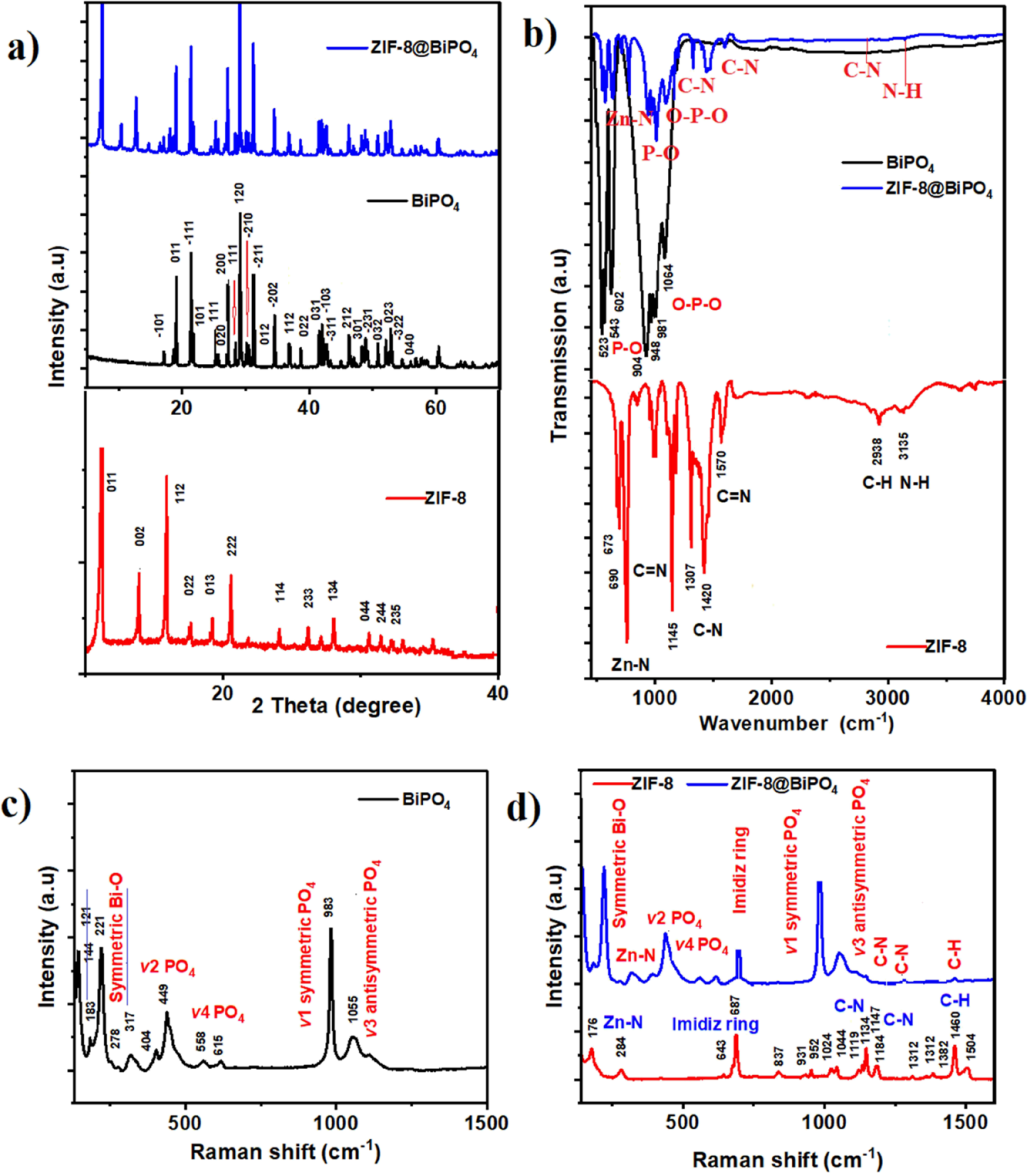
Structural characterization. (a) XRD patterns of ZIF-8 (red line), BiPO_4_(black line), and ZIF-8@BiPO_4_(blue line). (b) FTIR spectra of ZIF-8 (red line), BiPO_4_(black line), and ZIF-8@BiPO_4_(blue line). Raman spectrum of (c) BiPO_4_, (d) ZIF-8 (red line), and ZIF-8@BiPO_4_ (blue line).

Figure 2 presents the structural and chemical characterization of the synthesized hybrid material. To analyze the phase and structure of the as-prepared materials, X-ray diffraction (XRD) measurements were implemented. In Figure 2a (red line), the peaks at 2 theta values of 7.25, 10.44, 12.67, 14.71, 16.35, 18.01, 21.99, 24.50, 25.75, 29.68, 30.58, and 31.50° are referred to (011), (002), (112), (022), (013), (222), (114), (233), (134), (044), (244), and (235) planes of ZIF-8, respectively.^83^ In Figure 2a (black line), the peaks at 2 theta values of 17.04, 18.70, 21.32, 21.93, 25.33, 25.42, 27.25, 28.39, 29.17, 30.14, 30.57, 31.18, 34.5, 37.12, 38.69, 41.56, 42.18, 42.62, 43.05, 46.28, 46.98, 48.38, 48.98, 50.82, 51.96, and 53.18° referring (−101), (011), (−111), (101), (111), (120), (−210), (−211), (012), (−202), (112), (022), (031), (−103), (−311), (131), (212), (301), (231), (032), (023), (−322), and (040) planes of BiPO_4_.^84^ This result confirmed that as-synthesized BiPO_4_ has a monoclinic phase compatible with the 12ort he12re (JCPDS #15-0767). In Figure 2a (blue line), XRD results of the composite material ZIF-8@BİPO_4_ are shown in which all of the characteristic peaks of both ZIF-8 and BiPO_4_ can be detected obviously. The narrow and strong peaks indicate that the structure has highly crystalline properties.

The chemical configuration and chemical bonding of the samples were determined by applying FTIR. In Figure 2b (red line), the peak at 3135 cm^–1^ is related to the N–H stretching band of the 2–methyl–imidazole ligand. The bands belonging to methyl groups lie around 2938 cm^–1^ (imid-azole aliphatic C–H) and 1570 cm^–1^ (C–H stretching). The absorption bands between 1420 and 1307 cm^–1^ can be ascribed to C–N stretching, and the band located at 1145 cm^–1^ corresponds to C=N vibration. An axial deformity is apparent at 1570 cm^–1^ as the C=N band. The bands around 673 and 690 cm^–1^ can be related to Zn–N stretching. The results are compatible with the literature.^85^

In Figure 2b (black line), the bonds depicted around 523 and 602 cm^–1^ belong to symmetric stretching and asymmetric bending vibrations of P–O, and the occurred bonds between 904 and 1064 cm^–1^ can be confirmed to be the O–P–O linkage possessing BiPO_4_. Moreover, in Figure 2b (blue line) FTIR results of the composite material owing all of the characteristic peaks of ZIF-8 and BiPO_4_ are seen obviously.^86^

The Raman spectra of the materials are demonstrated in Figure 2c,d. In Figure 2c, the monitored peaks at 100–300 cm^–1^ belong to symmetric bending vibrations of Bi–O, and those around 558–615 cm^–1^ correspond to *v*_2_ and *v*_4_ bending vibrations of PO_4_ groups. Furthermore, the screened peaks at 983 and 1055 cm^–1^ are responsible for the *v*_1_ symmetric and *v*_3_ antisymmetric vibration modes of PO_4_ in BiPO_4_.^87^ In Figure 2d (red line), Raman spectra of ZIF-8 are shown. The peak of C–H is depicted at 1504 cm^–1^, the peaks between 1119 and 1184 cm^–1^ belong to C–N stretching, and the peaks around 643 and 952 cm^–1^ can be ascribed to the imidazolium ring.^88^ Additionally, the peak of Zn–N is depicted at 282 cm^–1^. Figure 2d (blue line) shows the Raman spectra of the composite material ZIF-8@BiPO_4_ including all characteristic peaks of raw materials. The peaks around 176–281 cm^–1^ belong to symmetric bending vibrations of Bi–O and those around 644–687 cm^–1^ correspond to *v*_2_ and *v*_4_ bending vibrations of PO_4_ groups, respectively. The depicted peaks at 984 and 1042 cm^–1^ are responsible for the *v*_1_ symmetric and *v*_3_ antisymmetric vibration modes of PO_4_ in BiPO_4_. Moreover, the peak of Zn–N is depicted at 284 cm^–1^. The broad peak around 649–952 cm^–1^ might be acceptable of the peaks of the imidazolium ring. Additionally, the peaks between 1147 and 1185 cm^–1^ refer to C–N stretching, and the peak at 1460 belongs to the C–H bond.

N_2_ adsorption–desorption isotherms were applied to scrutinize the porosity of the structures seen in Figure S1. In comparison with BiPO_4_, ZIF-8 and ZIF-8@BiPO_4_ display much more adsorbed amount of N_2_ gas, indicating the existence of micropores (Figure S1a). Pure ZIF-8 has the largest SSA-TPV 407.66 m^2^ g^–1^; 0.50771 cm^3^ g^–1^, while the SSA-TPV of BiPO_4_ and ZIF-8@BiPO_4_ is 2.79 m^2^ g^–1^; 0.00447 cm^3^ g^–1^, 275.73 m^2^ g^–1^; 0.35118 cm^3^ g^–1^, respectively. The addition of ZIF-8 enhanced the average pore size value of BiPO_4_; thus, BET results and pore sizes of ZIF-8@BiPO_4_ are higher than those of pure BiPO_4_. In Figure S1b, the pore size distribution of the patterns is demonstrated. The obtained average pore sizes of ZIF-8, BiPO_4_, and ZIF-8@BiPO_4_ are 11, 8, and 15 nm, respectively.

**Figure 3:**
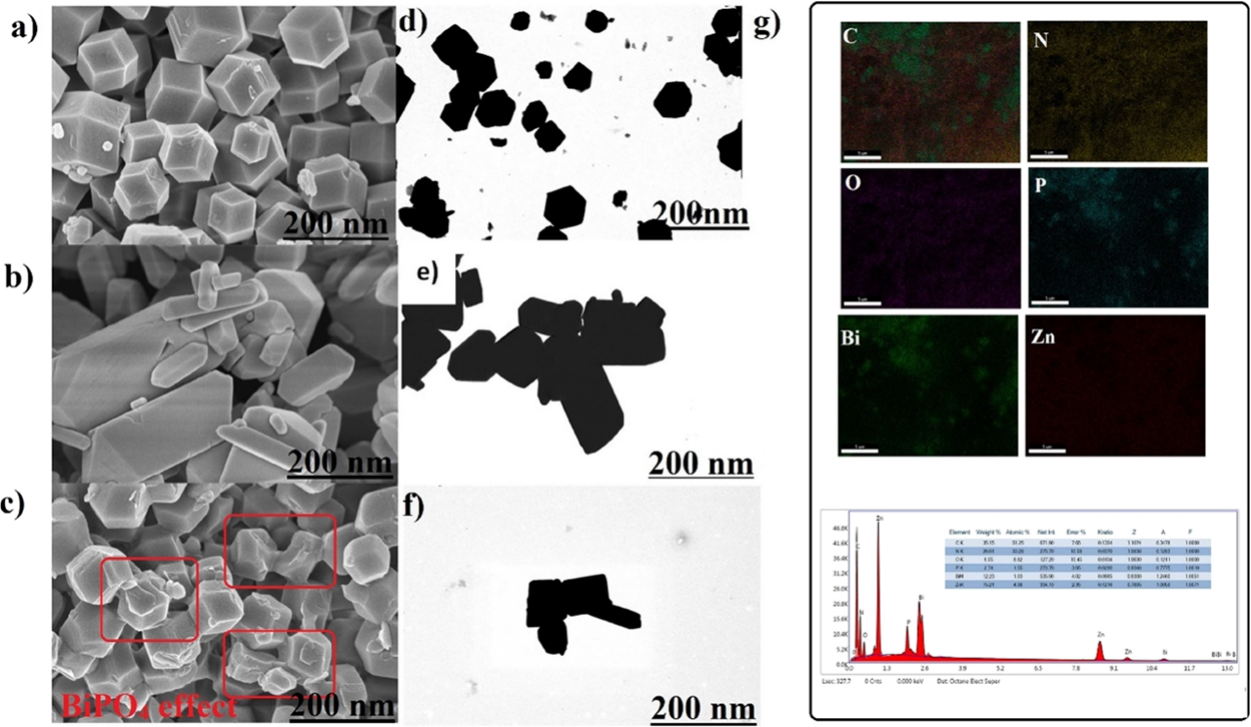
Electron microscopy images. (a–c) FESEM, and (d–f) STEM images of the samples (a–d) ZIF-8, (b–e) BiPO_4_, and (c–f) ZIF-8@BiPO_4_. (g) Individual elemental mapping image of C, N, O, P, Bi, and Zn, and spectra of EDS mapping, respectively.

In Figure 3, the surface morphology of the patterns screened by FESEM and STEM techniques is demonstrated. While ZIF-8 has a regular polyhedron shape (Figure 3a–d), BiPO_4_ mostly has irregular cubic-like shapes with agglomerated particles (Figure 3b–e). The composite ZIF-8@BiPO_4_ also shows (Figure 3c–f) both polyhedron and irregular agglomerated cubic-like morphology interwoven with each other. Energy-dispersive spectrometry (EDS) was utilized to identify the composition of the ZIF-8@BiPO_4_ structure. In Figure 3g, individual elemental mapping images of C, N, O, P, Bi, and Zn and EDS images are shown indicating that ZIF-8@BİPO_4_ combination was achieved successfully.

### 3.2 Electrochemical Studies

#### 3.2.1 CV Studies

**Figure 4:**
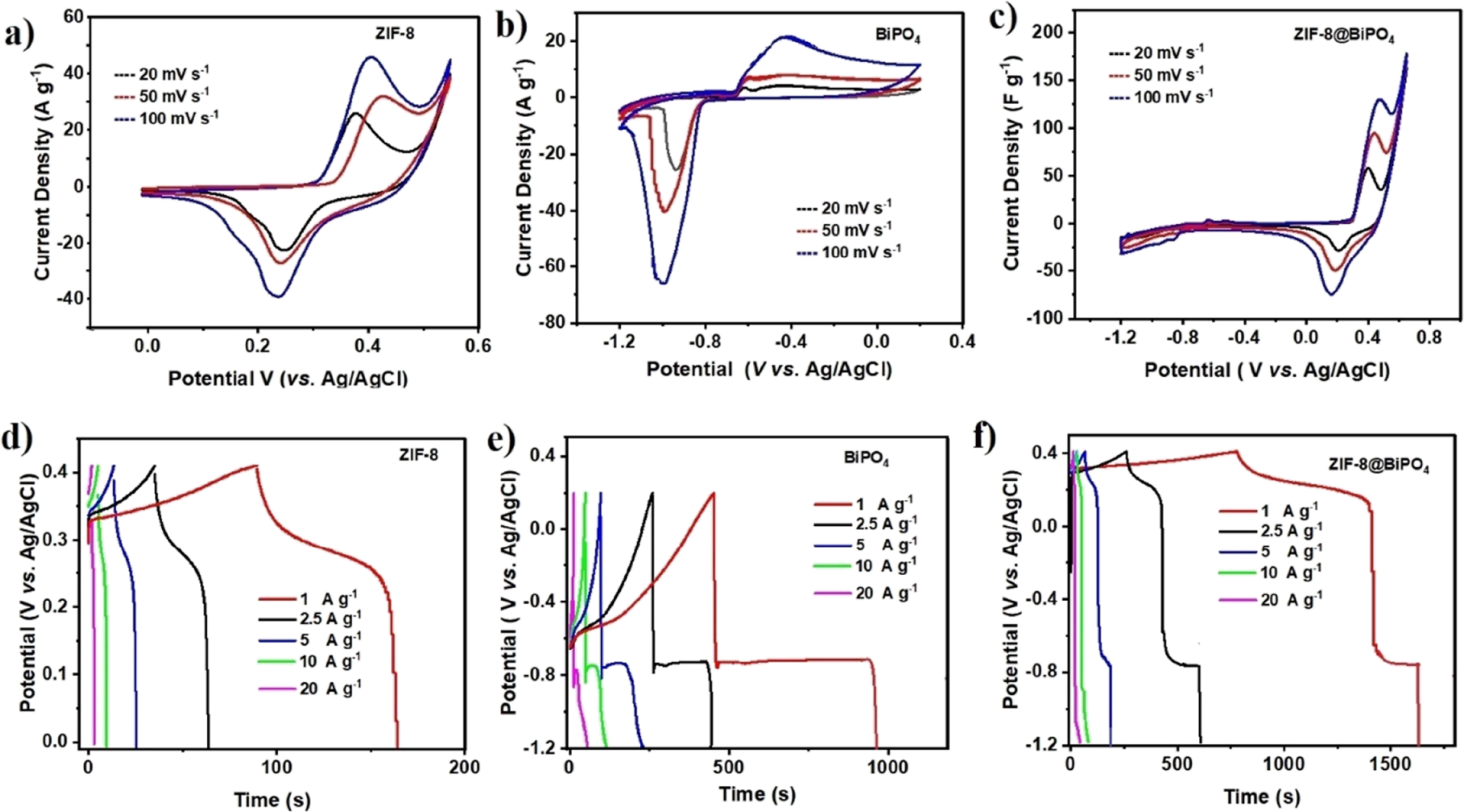
Cycling voltammograms of (a) ZIF-8, (b) BiPO_4_, and (c) ZIF-8@BİPO_4_. Charge–discharge curves of the electrodes at different current densities (d) ZIF-8, (e) BiPO_4_, and (f) ZIF-8@BiPO_4_.

The electrochemical characterization results are summarized in Figure 4. Figure 4a–c displays CV of the electrodes in a three-electrode system at different scan rates (20, 50, 100 mV s^–1^) in 2.0 M KOH (vs Ag/AgCl) electrolyte. ZIF-8 CV curves were examined in the potential range of −0.01 to 0.55 V (vs Ag/AgCl), while BiPO_4_ working potential was between −1.2 and 0.2 V (vs Ag/AgCl), and ZIF-8@BiPO_4_ CV curves were scanned between −1.2 and 0.65 V (vs Ag/AgCl). All of the materials have conspicuous redox peaks expressing the pseudocapacitive nature of the materials. By polarization with the scan rate increment, there is a shift toward the larger potential range on the anodic area while cathodic peak shift was toward the lower potential range.^22,89−91^ Regardless of the increment of the current density with the scan rate, all of the patterns yet had obvious redox peaks, which confirmed gratifying charge/discharge reversibility. The frequent redox reactions transpiring at the electrode/electrolyte interface conduce to redox peaks of the patterns to enhance with the increased scan rates.

The specific capacitance values of the electrodes at various scan rates were measured based on the following equation:
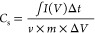
2

The area under the CV curves is designated by ∫*I*(*V*)Δ*t* , the scan rate is shown as *v* (mV s^–1^), *m* (g) presents the mass of the active material, and Δ*V* (V) typifies the applied potential window.

**Figure 5:**
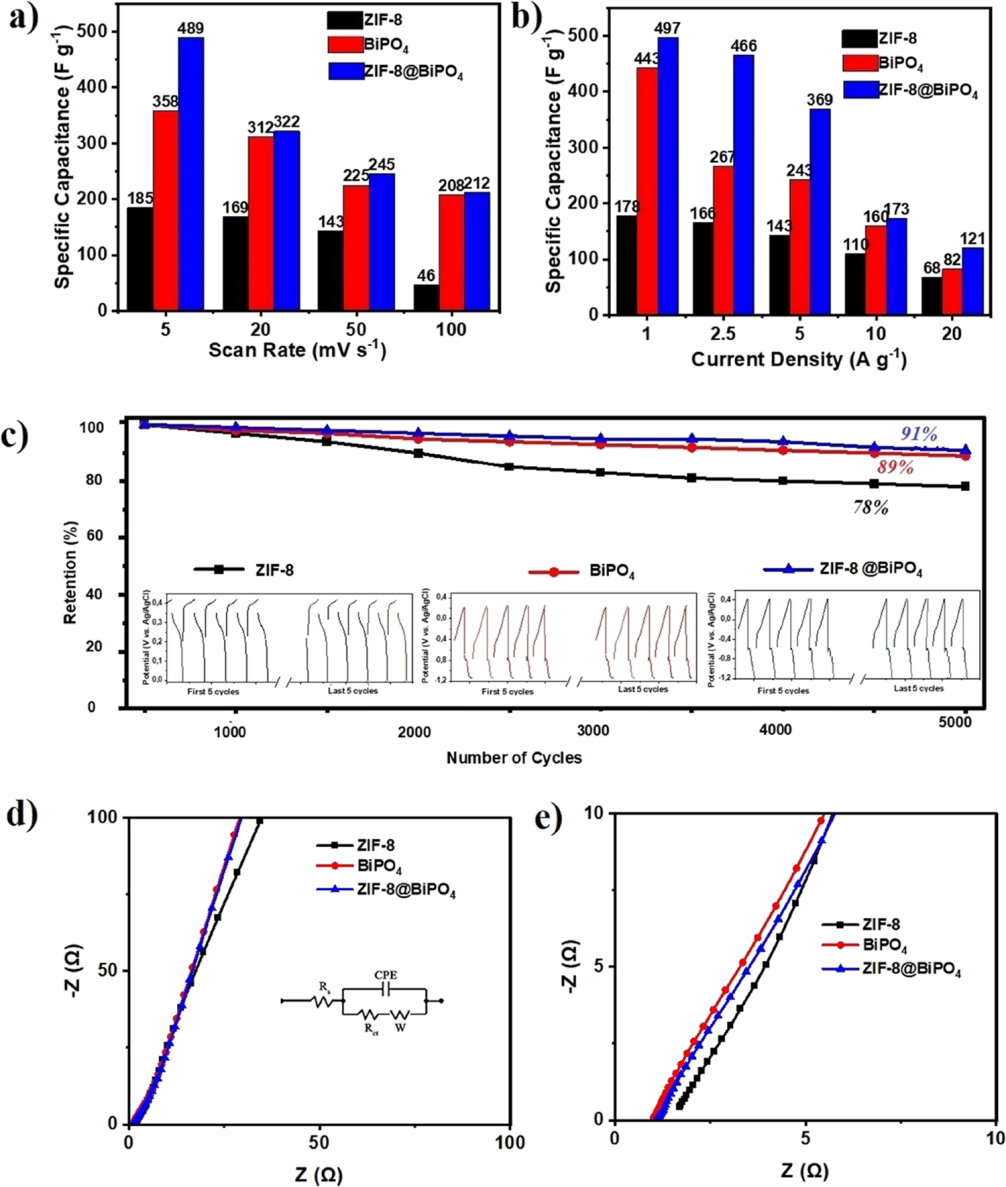
Specific capacitance values at different (a) scan rates and (b) current densities. (c) Comparison of cycling stability performance of the electrodes at 20 A g^–1^ current density, and individual charge–discharge curves showing first and last five cycles of the electrodes. Electrochemical impedance spectra of electrodes in low (d) and high (e) frequency ranges in 2 M KOH solution (inside corresponding equivalent circuit for modeling the measured impedance spectroscopy).

Due to the distinctness of the potential range of the electrodes, it was inconvenient to syllogize the obvious differences from CV graphs. However, the specific capacitances measured from eq 1 declared that composite ZIF-8@BiPO_4_ has the highest specific capacitance value of 489 F g^–1^ at a scan rate of 5 mV s^–1^.The maximum specific capacitance values of ZIF-8 and BiPO_4_ at the same scan rate are 185 and 358 F g^–1^, respectively. Although there were distinct differences from rising 5 to 20 mV s^–1^ for all electrodes, this disparity lessens with increasing scan rates as shown in Figure 5a. At high scan rates, there were not many differences between electrochemical features of pure BiPO_4_ and composite material ZIF-8@BiPO_4_.

#### 3.2.2 Chronopotentiometry

GCD measurements were applied to inquire about the charge–discharge behaviors and long-term cycling stability of the electrodes at different current densities (1–20 A g^–1^). The applicable potential window for ZIF-8 was −0.01 to 0.41 V (vs Ag/AgCl). The potential window of BiPO_4_ was the same with CV measurements as −1.2 to 0.2 V (vs Ag/AgCl), and the feasible potential window of ZIF-8@BiPO_4_ was −1.2 to 0.41 V (vs Ag/AgCl) in 2 M KOH aqueous electrolyte.

Due to more interaction occurring between the electrode and electroactive surface of OH^–^ ions, higher specific capacitance values can be obtained at low currents. However, the appeared diffusion effect causes a decrement in specific capacitance at high current densities.^12,92^

The specific capacitance of the electrodes was reckoned from the charge–discharge curves by the following equation.
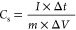
3where *I* (A) typifies the discharge current, Δ*t* (s) is the average discharge time, the mass of the active material abbreviated as *m* (g), and Δ*V* (V) represents the GCD applied potential window. The current density *J* (A g^–1^) was meant the ratio of discharge current *I* (A) to the mass of the active material *m* (g).

The individual charge–discharge curves of the electrodes at different current densities were graphed in Figure 4d–f. While ZIF-8 could reach a maximum specific capacitance of 178 F g^–1^ at a current density of 1 A g^–1^, the highest specific capacitances of BiPO_4_ and ZIF-8@BiPO_4_ were 443 and 497 F g^–1^ at the same current density, respectively. Although there were 0.2 V differences between BiPO_4_ (*V*_+_ = 0.2 V) to ZIF-8@BiPO_4_ (*V*_+_ = 0.4 V), the composite material still had higher specific capacitances than pure materials. In Figure 5b, the specific capacitance variations with current densities are depicted. Significantly, there was not a conspicuous discrepancy on specific capacitances between pure BiPO_4_ and ZIF-8@BiPO_4_ at high current densities such as 10 and 20 A g^–1^.

To further determine the long-term cycling stability of the electrodes long-term chronopotentiometry analysis was applied at a current density of 20 A g^–1^ up to 5000 cycles. In Figure 5c, the retained specific capacitance value of the electrodes is demonstrated. The retention of ZIF-8 (78%) was recorded more less than the retention of BiPO_4_ (89%). Satisfyingly, the capacity efficiency of ZIF-8@BiPO_4_ was higher than others (91%) indicating that the composite material has better cycling stability.

#### 3.2.3 Electrochemical Impedance Spectroscopy

To test the kinetics of ion and charge transfer action of the materials, EIS was performed. In Figure S2, individual Nyquist plots in low- and high-frequency ranges are shown. The comparison of EIS in low- and high-frequency ranges (inset displays the equivalent circuit model) in 2 M KOH solution is graphed in Figure 5d,e in the frequency range of 0.01–100 kHz with an amplitude of 5 mV.

When the ion diffusion resistance is determined by the low-frequency region, the high-frequency region is responsible for the charge transmission. The vertical line in the low-frequency area affirms the ideal capacity performance of the patterns. İon diffusion among the electrode and electrolyte causes another line called the Warburg line which makes 45°angle among that vertical line and the *x*-axis.

The charge transport resistance (*R*_ct_) of the electrodes ZIF-8, BiPO_4_, and ZIF-8@BiPO_4_ was 0.80, 0.56, and 0.76 mΩ, respectively. The equivalent series resistance (*R*_s_) for the electrode BiPO_4_ (1.04 Ω) was lower than that of ZIF-8 (1.72 Ω), although there was not a noticeable discrepancy between BiPO_4_ (1.04 Ω) and ZIF-8@BiPO_4_ (1.16 Ω). As declared, BiPO_4_ has the minimum and ZIF-8 has the highest values of *R*_ct_ and *R*_s_.

#### 3.2.4 Electrochemical Performances of an ASC

To further test the operation of the electrodes in practical applications, a two-electrode system ASC was fabricated by using ZIF-8@BiPO_4_ as the cathode and BiPO_4_ as the anode material with a glass microfiber filter separator presoaked in 2 M KOH. The optimal mass balance of anode and cathode electrodes (*M*_+_/*M*_–_) was calculated to be ∼1.3 by the following equation.^93−95^
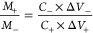
4where *M* represents the mass of the active material, *C* symbolizes specific capacitance, and Δ*V* signifies the potential window for the anode and cathode electrodes.

**Figure 6:**
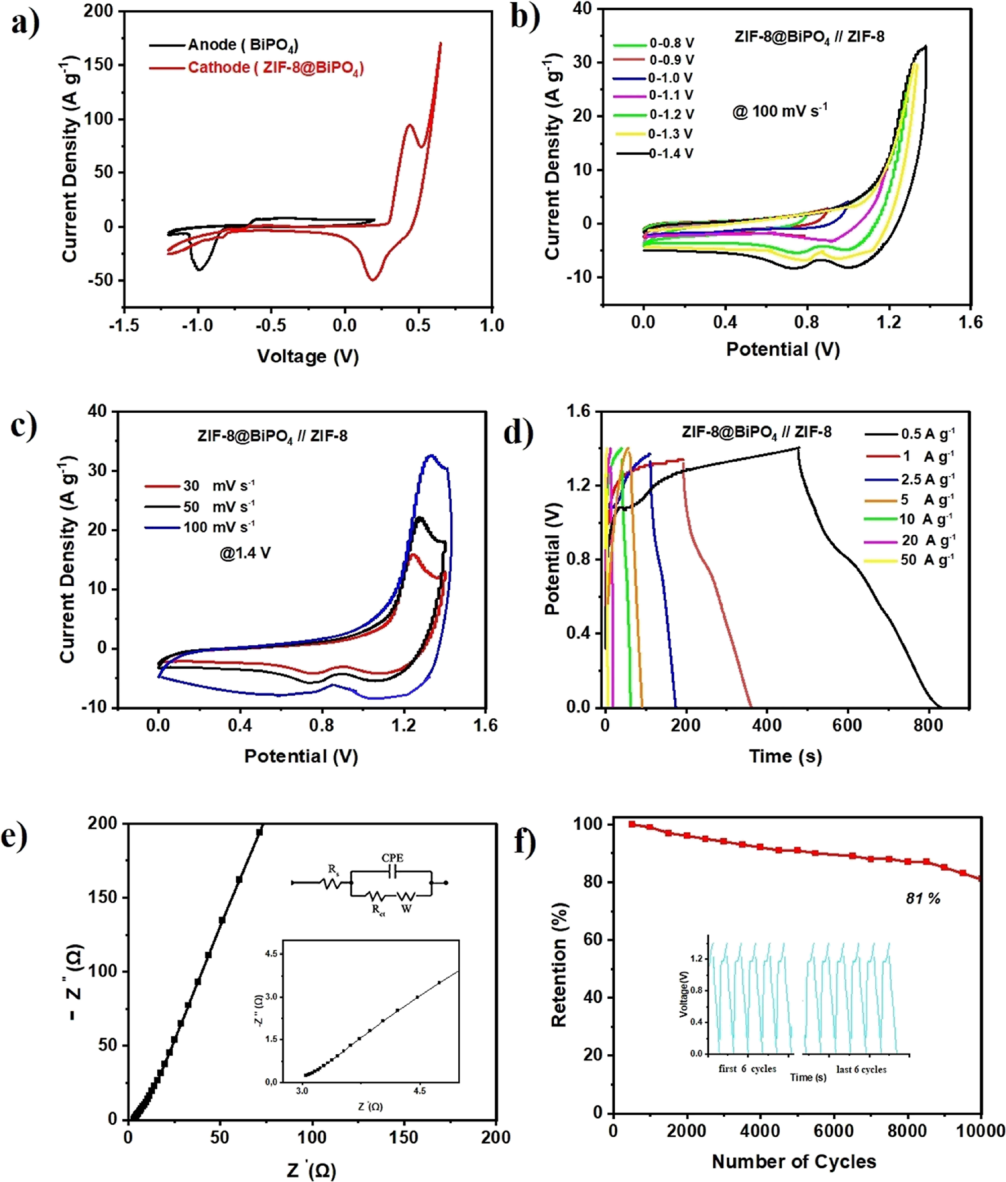
Electrochemical performance of the ASC device. (a) Individual CV curves of anode and cathode materials, (b) CV curves of the ASC device in various potential ranges, (c) CV curves of ASC device at different scan rates, (d) GCD profile of the ASC device at various current densities, (e) EIS of the ASC device in low- and inside high-frequency ranges, and (f) cycling stability and capacitance retention of the ASC device at a current density of 10 A g^–1^.

In Figure 6, electrochemical features of the ASC device are illustrated. Figure 6a shows individual CV curves of anode BiPO_4_ [−1.2 to 0.2 V (vs Ag/AgCl)] and cathode ZIF-8@BiPO_4_ [−1.2 to 0.65 V (vs Ag/AgCl)]. Figure 6b demonstrates the CV curves of the ASC device in different potential windows ranging from 0.8 to 1.4 V at a scan rate of 100 mV s^–1^. The determined suitable potential window was 0–1.4 V. CV curves of the ASC device in the cell voltage limit of 1.4 V at the different scan rates (30–50–100 mV s^–1^) are also shown in Figure 6c. At both cathodic and anodic scans, the reduction and oxidation peaks can be detected, and these peaks are more conspicuous at lower scan rates. This could be predicated on the deintercalation process that appears at the surface of the electrodes.

The specific capacitance (*C*_sp_, F g^–1^) of the ASC cell, the energy density (*E*, Wh kg^–1^), and the power density (*P*, W kg^–1^) were reckoned up according to the following equations:
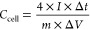
5
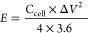
6
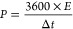
7

**Figure 7:**
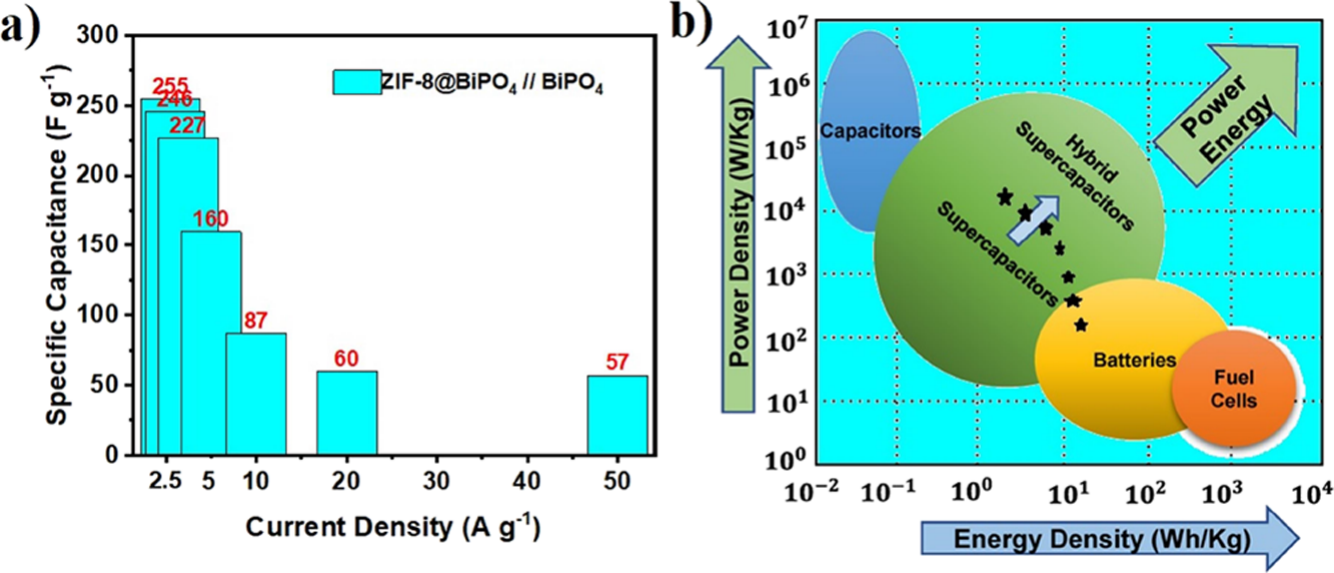
(a) Schematic illustration of the specific capacitance results according to current densities, and (b) Ragone plot of the ASC.

The GCD behavior of the ASC device at various current densities is shown in Figure 6d. Well-proportioned charge and discharge curves indicate a fast *I*–*V* reaction. The calculated maximum and minimum specific capacitance values by using eq 5 were 255 F g^–1^ at a current density of 0.5 A g^–1^ and 57 F g^–1^ at a current density of 50 A g^–1^ as illustrated in Figure 7a.

To check the kinetics of ion and charge transfer reaction of the ASC device, EIS analysis was employed. The Nyquist plot of the ASC was graphed in Figure 6e in the frequency range of 0.01–100 kHz at open circuit potential. The obtained *R*_s_ and *R*_ct_ values are 2.87 Ω and 6.91 mΩ, respectively.

Additionally, to test the long-term cycling stability of the ASC device, a constant current density of 10 A g^–1^ was applied up to 10,000 cycles as shown in Figure 6f. The retention of the specific capacity of the ASC device was 81% of its initial capacity after 10,000 cycles, indicating that the formed ASC device shows good cycling stability.

The connection between energy and power densities of the ACS is demonstrated in the Ragone plot in Figure 7b. For the fabricated ASC device, the obtained maximum energy density was 17.5 Wh kg^–1^ at a power density of 178 W kg^–1^ and a boosted power density of 13,695 W kg^–1^, giving an energy density of 2.9 Wh kg^–1^. The detailed results are tabularized in Table 1.

**Table 1:** Energy and Power Densities of the ASC at Different Current Densities

energy density/power density (E/P) (Wh kg^–1^/W kg^–1^)							
current density	0.5 A g^–1^	1 A g^–1^	2.5 A g^–1^	5 A g^–1^	10 A g^–1^	20 A g^–1^	50 A g^–1^
energy density	17.5	16.5	15	10.85	6	4	2.9
power density	178	355	882	1866	3071	7067	13,695

#### 3.2.5 Photocatalytic Activity Evaluation of ZIF-8@BiPO_4_

**Figure 8:**
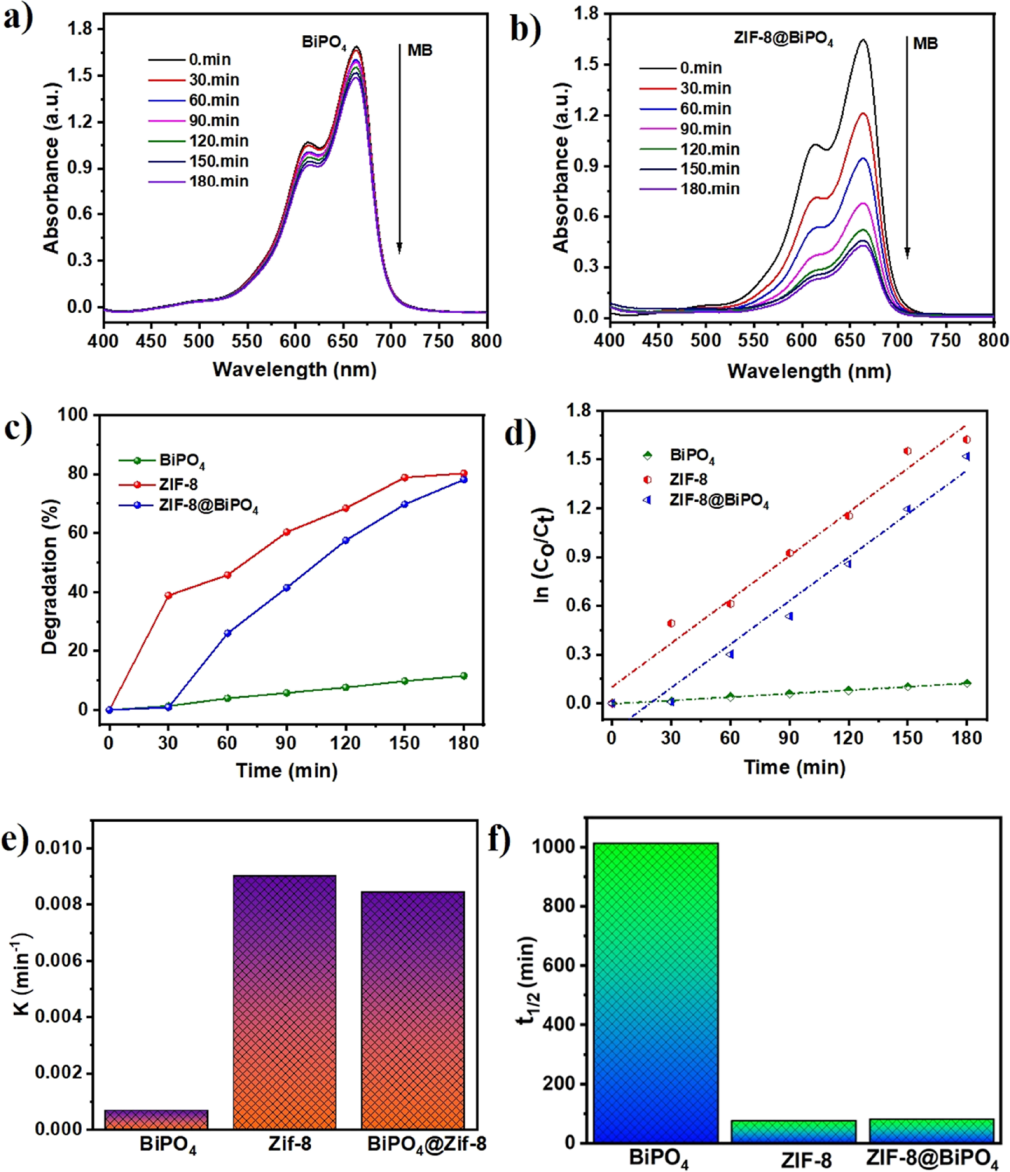
Photocatalytic activity of BiPO_4_, ZIF-8, and ZIF-8@BiPO_4_ hybrid. UV–vis absorbance of aqueous MB solution withdrawn on (a) BiPO_4_ and (b) ZIF-8@BiPO_4_, over a time interval ranging from 0 to 180 min. (c) Photocatalytic degradation (%) of MB on BiPO_4_, ZIF-8, and ZIF-8@BiPO_4_ (*t* = 180 min). (d) Plot of photocatalytic activity of BiPO_4_, ZIF-8, and ZIF-8@BiPO_4_ as a function of time. (e) Pseudo-first-order reaction rate constant. (f) Half-life time of the photocatalytic degradation rate.

The photocatalytic activity of the synthesized ZIF-8@BiPO_4_ hybrid material was evaluated by performing the degradation of the MB dyestuff and sildenafil citrate selected as the probe molecules under UV light. In the evaluation of the photocatalytic activity of ZIF-8@BiPO_4_ hybrid material, the decrease in the intensity of the absorption peak given by the MB dyestuff at 664 nm as a function of the increasing photocatalytic reaction time was used. Figure 8a,b shows the UV–vis spectra of the photocatalytic degradation of 10 mg L^–1^ MB dyestuff under UV light, containing 1 mg mL^–1^ of BiPO_4_ and ZIF-8@BiPO_4_ nanomaterials, respectively. In experiments carried out in model solutions containing 1 mg mL^–1^ BiPO_4_, ZIF-8, and ZIF-8@BiPO_4_ nanophotocatalysts, respectively, it was observed that MB dyestuff was removed with approximately 15, 81, and 78% efficiency within 180 min (Figure 8c). To ensure that the adsorption process in the mixing processes carried out in the dark was completed before the photocatalytic reaction started, it should be noted that the ZIF-8 nanomaterial exhibited the best adsorption properties and therefore had the highest adsorption capacity. The photocatalytic activity of the ZIF-8@BiPO_4_ nanophotocatalyst was lower than that of pure ZIF-8. This is because when BiPO_4_ nanoparticles accumulate on the surface of ZIF-8 nanoparticles, it blocks the active sites of ZIF-8 and reduces the SSA of ZIF-8. As a result, the contact area between ZIF-8 nanoparticle particles and MB dyes will decrease, resulting in a decrease in photocatalytic performance (Figure 8d).

To evaluate the reaction kinetics of MB degradation, the degradation data were fitted by using the pseudo-first-order chemical kinetics reaction model (eq 8) and expressed by the following eq 9:
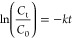
8where *k*, *t*, *C*_0,_ and *C*_t_ are the kinetic constant, time, initial equilibrium concentration of MB, and the concentration of MB at the given time “*t*” min, respectively.
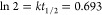
9

The first-order reaction rate constant (*k*, min^–1^) and the half-life time (*t*_1/2,_ min) of BiPO_4_, ZIF-8, and ZIF-8@BiPO_4_ nanophotocatalysts were found to be 0.00068 min^–1^ and 1013 min, 0.00901 min^–1^ and 77 min, and 0.00844 min^–1^and 82 min, respectively (Figure 8e,f).

The same photocatalytic experiments explained above were carried out for degradation of CS. In the evaluation of the photocatalytic activity of the ZIF-8@BiPO_4_ hybrid material, the decrease in the intensity of the specific absorption peak for SC on the UPLC-DAD analysis system as a function of the increasing photocatalytic reaction time was used. The obtained results given in Figure S3 show that CS was removed with approximately 100% efficiency within 420 min.

To illuminate the photocatalytic process of the Zif-8@BiPO_4_ hybrid material, the UV–vis spectrum of Zif-8, BiPO_4_, and Zif-8@BiPO_4_ was measured, and the Tauc plot was applied to the determine band gaps by using the obtained UV–vis spectra (Figure S4). It was found that Zif-8, BiPO_4_, and Zif-8@BiPO_4_ have a maximum absorption peak centered at 360, 330, and 355 nm wavelengths, respectively. As the band gaps for Zif-8 and BiPO_4_ were calculated as 2.80 and 3.41 eV, the band gap for Zif-8@BiPO_4_ was found as 2.40 and 3.87 eV. ZIF-8 and BiPO_4_ demonstrate a narrow and wide band gap, respectively, and Zif-8@BiPO_4_ exhibits a slightly wider band gap compared with the directly generated BiPO_4_ sample due to the movement of the O_2p_ state at the top of the valence band to the low-energy direction caused by the produced O_vac_.

Reusability is an important feature to evaluate the stability of catalysts. The reusability performance of Zif-8@BiPO_4_ hybrid catalysts was evaluated using MB dyestuff with repeated uses. Zif-8@BiPO_4_ hybrid catalysts did not perform consistently over three cycles of photodegradation. After three cycles, the photocatalytic reusability of the hybrid catalysts decreased significantly. It has been determined that the decrease in the degradation value obtained at the end of the third cycle is about 30% (1st cycle: 78% and 2nd cycle: 54%).

## 4 Conclusions

To conclude, we have developed a hybrid material by synergetic integration of ZIF-8 and BiPO4 nanoparticles for addressing the pressing challenges in energy storage and water remediation applications. The electrochemical features of the formed electrodes were optimized in 2 M KOH electrolyte in a three-electrode cell configuration. The Cs of hybrid material ZIF-8@BiPO_4_ (489 F g^–1^ at a scan rate of 5 mV s^–1^; 497 F g^–1^ at a current density of 1 A g^–1^) was substantially higher than that of pristine materials BiPO_4_ (358, 443 F g^–1^) and ZIF-8 (of ∼185, 178 F g^–1^) under the same conditions. The ASC device fabricated by using BiPO_4_ as the anode and ZIF-8@BiPO_4_ as cathode electrodes received an excellent Cs of 255 F g^–1^ at a current density of 0.5 A g^–1^. The cycling stability of the ASC was also as high as 81% over 10,000 cycles. Additionally, the acquired highest energy and power densities were 17.5 Wh kg^–1^ and 13,695 W kg^–1^, respectively. Thereafter, the degradation of MB was examined for the use of photocatalysis implementation. After 180 min of UV irradiation, the degradable amount of MB was obtained as 78%. These results show that the as-prepared environmentally kind, reasonably priced, multifunction hybrid material can find an execution area on supercapacitors and photocatalytic applications.

## Supporting Information Available

N_2_ adsorption–desorption isotherms and the pore size distribution of the patterns, electrochemical impedance spectra of the electrodes, photocatalytic degradation (%) of sildenafil citrate, and UV–VIS spectra and the corresponding Tauc plot of all photocatalysts (PDF)
