# Supplementary material for: Rational Integration of ZIF-8 and BiPO4 for Energy Storage and Environmental Applications
Source: ACS Omega. 2022 Nov 28;7(49):44878–91. doi: 10.1021/acsomega.2c04835 (PMC9753177; doi:10.1021/acsomega.2c04835)
Supplement: Supplementary file 1 — ao2c04835_si_001.pdf [file ao2c04835_si_001.pdf]

SUPPORTING INFORMATION

Rationale integration of ZIF-8 and $BiPO_4$ for Energy Storage and Environmental Applications

Sevda Yetiman[a], Sultan Karagoz[a,b], Fatma Kilic Dokan [c,*], M. Serdar Onses [a, d], Erkan Yilmaz [a,e ,g], Ertugrul Sahmetlioglu [a,f,*]

[a] ERNAM-Erciyes University Nanotechnology Application and Research Center, Kayseri, 38039, Turkey

[b] Department of Textile Engineering, Faculty of Engineering, Erciyes University, Kayseri, 38039, Turkey

[c]Department of Chemistry and Chemical Processing Technologies, Mustafa Çıkrıkcıoglu Vocational School, Kayseri University, Kayseri, Turkey

[d]Department of Materials Science and Engineering, Faculty of Engineering, Erciyes University, Kayseri, 38039, Turkey

[e]Technology Research & Application Center (TAUM), Erciyes University, Kayseri, 38039, Turkey

[f]Department of Basic Sciences of Engineering, Kayseri University, Kayseri, 38039, Turkey

[g]Department of Analytical Chemistry, Faculty of Pharmacy, Erciyes University, Kayseri, Turkey

*Corresponding authors ; fatmakilic@kayseri.edu.tr, sahmetlioglu@erciyes.edu.tr

N_2 adsorption-desorption isotherms and the pore size distribution of the patterns

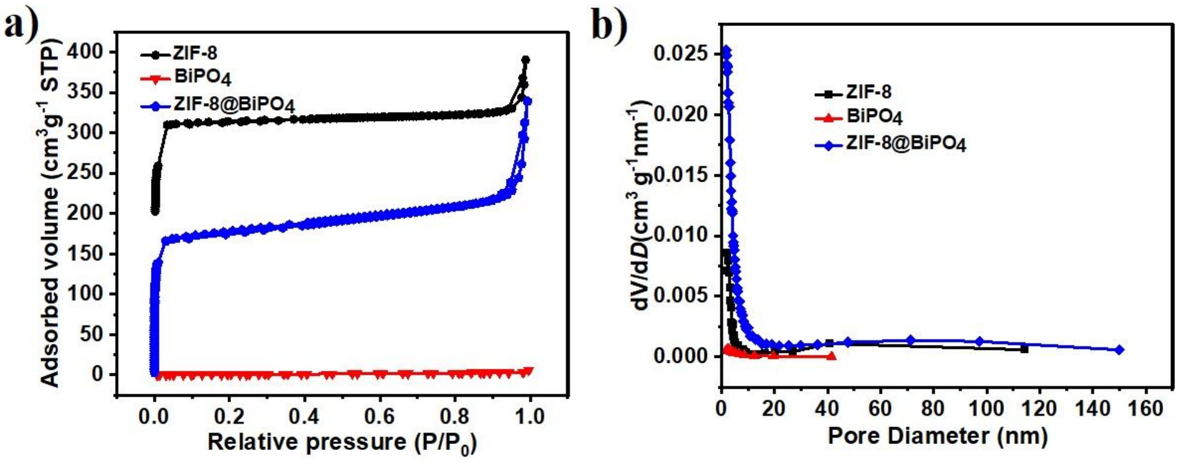

Figure S1. (a) N_2 adsorption-desorption isotherms of ZIF-8 (black line),$BiPO_4$ (red line),and ZIF-8@ $BiPO_4$ (blue line),(b) The pore size distribution of the patterns

Electrochemical impedance spectra (EIS) of the electrodes

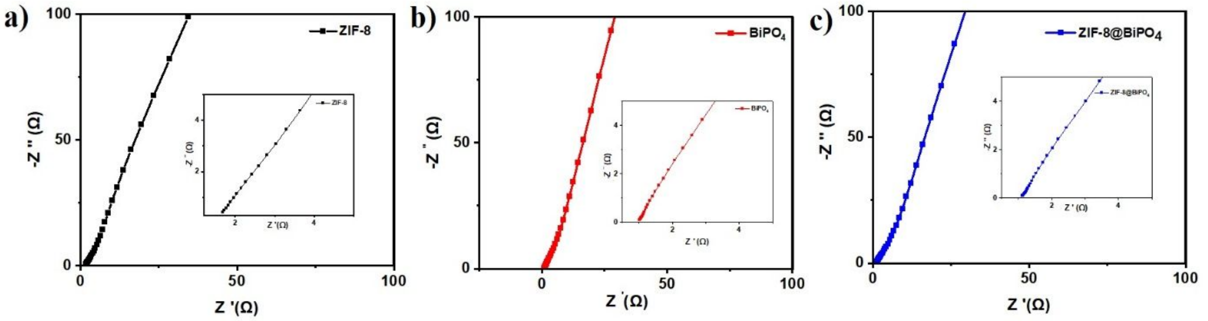

Figure S2. Electrochemical impedance spectra (EIS) of (a) ZIF-8 ,(b) $BiPO_4$,(c) ZIF-8@$BiPO_4$ electrodes at low and high frequency range in 2 M KOH solution .

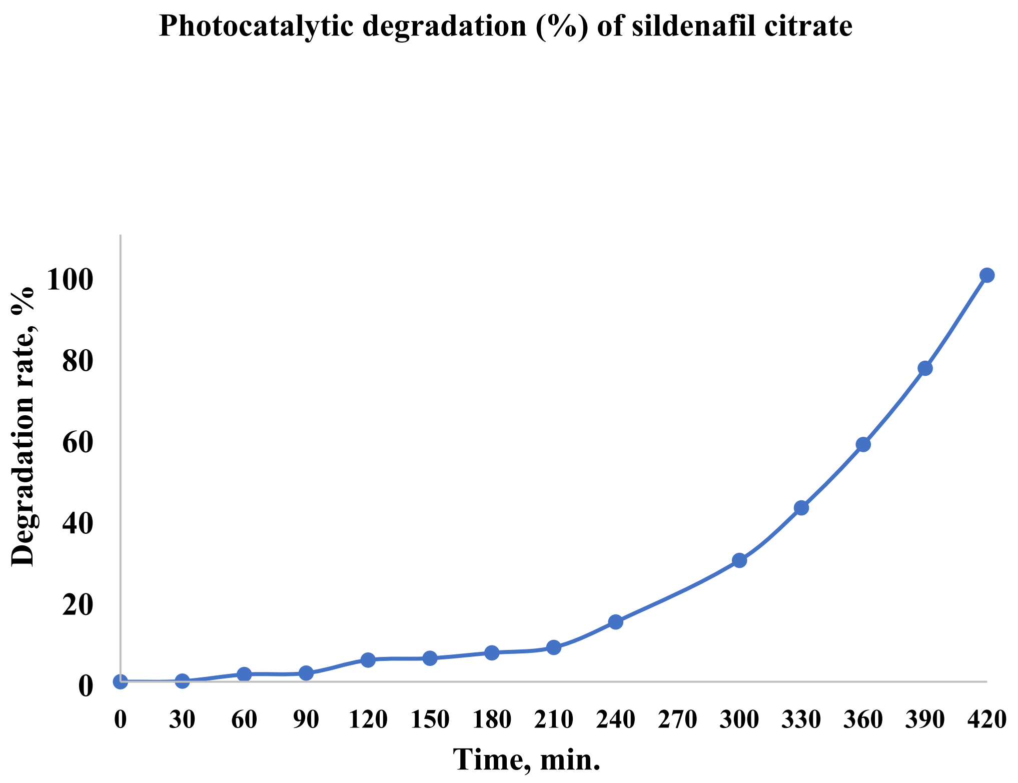

Figure S3. Photocatalytic degradation (%) of SC on ZIF-8@$BiPO_4$(t =420 min).

UV-VIS spectras and the corresponding Tauc plot of all photocatalysts

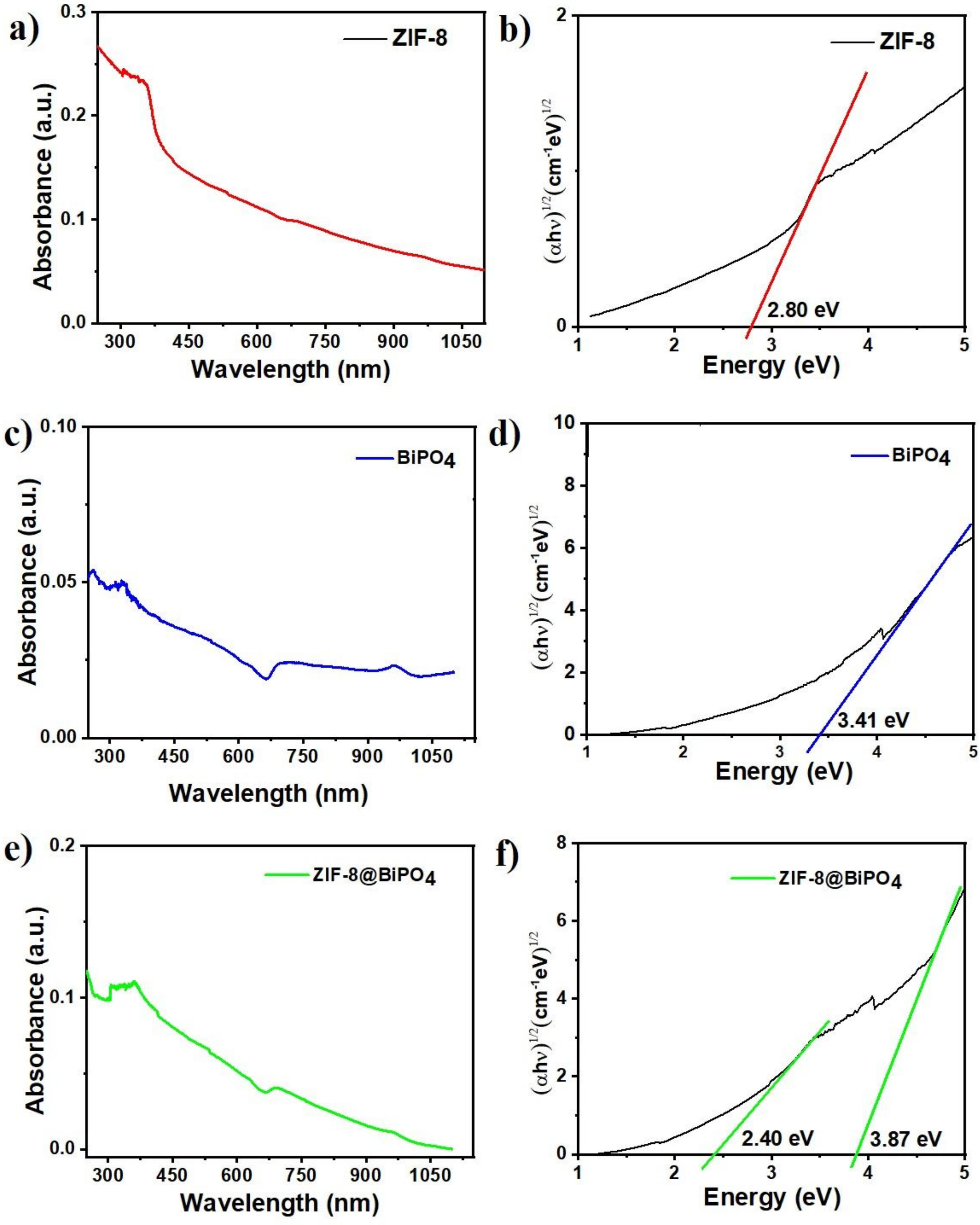

Figure S4. (a- c- e) UV-VIS spectras of all photocatalysts and (b- d- f) the corresponding Tauc plot of all photocatalysts
